# Examining Ethiopia's live animal and meat value chain

**DOI:** 10.1016/j.heliyon.2025.e41752

**Published:** 2025-01-06

**Authors:** Asrat Ayza Wakaso, Yesihak Yusuf Mummed, Yonatan Kassu Yesuf

**Affiliations:** a Department of Animal Science, Wolaita Sodo University, P.O.BOX. 138, Wolaita Sodo, Ethiopia; b School of Animal and Range Sciences, Haramaya University, P.O. BOX 135, Dire Dawa, Ethiopia

**Keywords:** Meat animals, Value chain, Transaction efficiency, Ethiopia, Market challenges

## Abstract

This review examines the efficiency of live animal and meat value chain from producers to consumers in Ethiopia. Ethiopia has a large livestock population, but the marketing system for live animals and meat remains underdeveloped. Several challenges hinder efficient transactions, including poor infrastructure, illegal cross-border trade, lack of market information, traditional production methods, and absence of grading systems. As a result, producers often receive low prices and have limited access to export markets. The key actors in the value chain, are input suppliers, farmers, traders, cooperatives, exporters, abattoirs, and consumers. However weak linkages and lack of coordination among these actors lead to inefficiencies. The trends in Ethiopia's livestock exports have fluctuated, with live animal exports exceeding meat product exports due to supply constraints and inability to meet quality standards for processed meat. The review highlights opportunities to strengthen the value chain through infrastructure upgrades, improved market information systems, promoting quality standards, and aligning production with export requirements. Coordinated efforts involving the government, private sector, and development partners are needed to address the challenges and unlock the potential of Ethiopia's meat animal value chain for the benefit of producers, traders, consumers, and the overall economy.

## 1 Introduction

Livestock production is an integral part of Ethiopia's agriculture and plays a pivotal role in national economic development. The livestock sector has enormous potential, with the largest cattle population in Africa at over 60 million heads [1] and contributor to the livelihoods of the rural poor in developing countries through important economic and traditional activities. They are raised to provide traction or produce commodities such as meat, milk, and eggs, and not only contributes to the agricultural GDP (45 %) and the overall GDP (19 %), but also plays a significant role in foreign exchange earnings, accounting for 16–19 % [2]. Additionally, the livestock sector provides employment to over 30 % of the agricultural labor force. The country produces over one million tonnes of beef valued at USD 2.5 billion, 3.8 billion liters of milk valued at USD 5.1 billion, and 116 million eggs [3].

However, the sector operates far below its potential, with average carcass weights of 110 kg versus 143 kg/head in East African countries or the continental average (156 kg/head) and 212 kg globally [4,5]. Similarly, beef cattle productivity is just 0.25 kg/day compared to 1.5 kg/day in other developing regions [6]. Consequently, per capita annual meat consumption is only 9 kg, compared to 25 kg in developing nations and 77 kg in developed countries [3]. For achieving sustainable development goals (SDGs), particularly goals 1, 2, and 12, which are about eliminating poverty, zero hunger, and responsive and sustainable production and consumption, is crucial for ensuring food and nutrition security, as well as environmentally responsible beef production [7].

Despite livestock's vital role in the livelihoods of smallholder farmers and pastoralists, Ethiopia's production systems remain largely subsistence-based rather than market-oriented [8]. Both legal and illegal marketing channels operate, dominated by small-scale transactions driven by household cash needs rather than commercial interests [9]. In formal live animal exports, cattle account for approximately 70 % of volumes, while meat exports derive almost entirely from small ruminants like sheep and goats [8].

According to Rozina [10], underdeveloped value chains pose significant threats to productivity and profitability across Ethiopia's livestock sector. The system involves a diverse array of actors such as feed providers, farmers, fatteners, brokers, wholesalers, retailers, end consumers, and multi-staged service providers. However, weak linkages and misalignment among these nodes engender inefficiencies and lost opportunities [11].

Agricultural value chains aim to enhance productivity by improving coordination from input supply through to final consumption and value addition [12]. While current knowledge on structure, performance and price transmission across Ethiopia's meat value chain remains inadequate hindering policy reforms [11]. Therefore, this review assesses efficiency and prevailing challenges in Ethiopia's meat value chain, in order to identify strategies to unlock the sector's productivity potential. Enhancing understanding of constraints and opportunity points will enable evidence-based interventions to optimize benefits for producers, traders, consumers and the wider economy.

## 2 Review method

This comprehensive narrative review evaluates the efficiencies and challenges present within Ethiopia's live animal meat value chain, with a particular focus on production systems, market dynamics, infrastructure, grading and certification standards, policy frameworks, and export opportunities. The study used a systematic approach to identify, synthesize, and analyze relevant data from peer-reviewed journals, legal briefs, and reputable indexed databases, including Google Scholar, Web of Science, PubMed, and Scopus. Employing targeted keywords such as “Ethiopian meat value chain,” “beef cattle production,” “livestock market inefficiencies,” “grading and certification systems,” and “meat export potential,” the research ensured a focused and comprehensive literature search. Studies published in the last decade were prioritized, with inclusion criteria emphasizing empirical findings and theoretical insights, while irrelevant studies were excluded.

The extracted data were thematically organized into seven key areas: livestock production systems, market structures, infrastructure adequacy, grading practices, regulatory frameworks, and trends and prospects in live animal exports. A rigorous cross-referencing strategy was applied to validate the findings and ensure their credibility. This review identified systemic inefficiencies by synthesizing thematic insights and proposed evidence-based strategies for sustainable improvement.

## 3 Literature review

### 3.1 Meat animal transaction along the value chains in Ethiopia

The meat animal and meat value chain links consumers to farmers and offers an opportunity to integrate smallholder producers into modern markets, both domestically and internationally [13]. Every value chain has a coordination system that includes both formal and informal arrangements between the participants. Coordination structures may range from loosely coordinated to strongly coordinated and integrated production and marketing systems [8,11].

The promise and potential of the Ethiopian livestock value chain is to become a thriving industry that can produce packaged meat destined for Middle Eastern, European, and East African markets or fashion gloves and shoes that sell in volume on the high streets and boutiques of Europe [14].

To reach this level of growth and development, operators and investors along the value chain may consider ways to improve the quality and value of meat exports by establishing a standardized grading system for meat and live animals, encouraging more supply into abattoirs to increase capacity utilization, thereby lowering costs, improving cost competitiveness, providing more raw material for leather producers, and introducing proper and improved feeding, fattening, animal health care, and other services, while encouraging foreign and domestic investment at all points along the value chain [14,15].

Although Ethiopia is among the few African countries exporting live animals on hooves and chilled sheep and goat carcasses to the Gulf States and North African countries, the export volume to Middle Eastern countries is low compared to other competitors, considering the resource base and other comparative marketing advantages [16]. The country earned $68.4 million from meat exports in the year 2019/20 and $32.1 million in 2020/21 for the half year. The major export markets for Ethiopian sheep and goat carcasses are the United Arab Emirates (52 %) and the Kingdom of Saudi Arabia (41 %). The remaining 7 % of meat is exported to Hong Kong, Vietnam, Qatar, and other countries. In 2019/20, new export market destinations emerged in East Asia [17]. In addition, neighboring countries such as Sudan (19.5 %), Somalia (19.0 %), and Djibouti (14.9 %) are the major live animal importers in Ethiopia [18].

According to the Ethiopian Meat Producers and Exports Association's March 2021 data, there are fourteen export abattoirs with approximately 200,000 tons of sheep, goats, and beef produced per year. However, existing meat processing facilities operate at less than 10 % of the full capacity because export abattoirs are unable to procure a suitable quality and quantity of live animals for the export market, and there is a high demand for meat in local markets. Hence, export abattoirs compete with the insufficient domestic supply of live cattle and shoats [17].

The Ethiopian export market for live animals and meat has shown fluctuations, with an overall declining trend, especially in live animal exports [10]. Non-standardized transactions exist across supply chains. Traders purchasing animals by visual estimation at local markets must sell by weight at export abattoirs, creating profit uncertainty [19].

To minimize risk, these collectors restrict purchase volumes, manifesting in supply shortages for exporters [8]. Producers ultimately lose out as traders avoid risk [11]. This discourages pastoralists from supplying export markets, as small traders purchasing by eyeball estimations eventually sell to abattoir agents using scales [20]. Thus, standardized transaction systems are needed across livestock markets to encourage market participation and bolster export competitiveness [[12], [21], [22],].

**Fig. 1:**
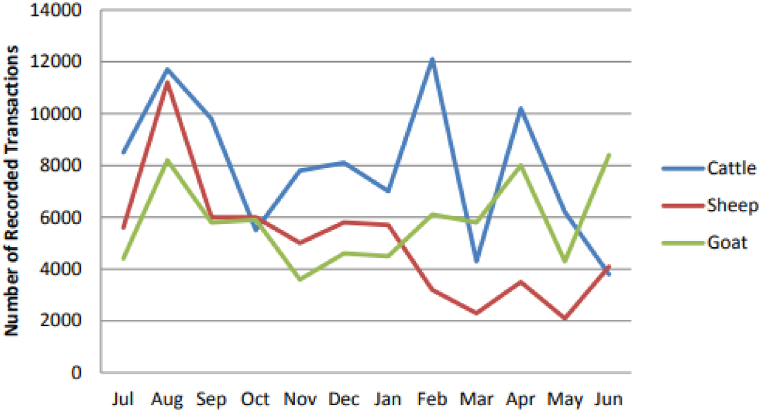
Livestock transactions from selected areas for cattle and shoats.

Source: AGP-LMDP [23].

Uniform purchase and sales methods can reduce risks and uncertainty for actors across the chain. For example, the chart below (Fig. 1) shows the livestock transactions from selected areas, including Metharam, Babile, Yabello, and Dubuluq of Ethiopia [23].

According to MoA and ILRI [18] stated that several factors may influence the volume and direction of flow of live animals from production areas to various destinations, such as costs of transport, feed or forage availabilities, trekking routes, policies, institutions, infrastructure, and prices. Livestock sales decisions by farming households, particularly by pastoralists, are basically a function of their basic needs, such as food grains, clothing, health care, and a period of drought.

### 3.2 Meat animal value chains

Unlike other commodities, the value chains for livestock and their products consist of diverse actors with easily identifiable and distinct transactions. The marketing of these products entails significant risks and high maintenance, transportation costs, and involves various stakeholders [8]. As a result, the concept of the agricultural value chain encompasses all activities and participants involved in moving agricultural products from input suppliers to farmers' fields and ultimately to consumers. Each stakeholder in the value chain contributes to the formation of a cohesive and viable system [10,12]. By examining the complete production to consumption system of cattle and shoats in the terminal market of Addis Ababa, it is possible to identify how marketing and value-addition activities occur and determine the distribution of benefits generated by such activities [24].

**Fig. 2:**
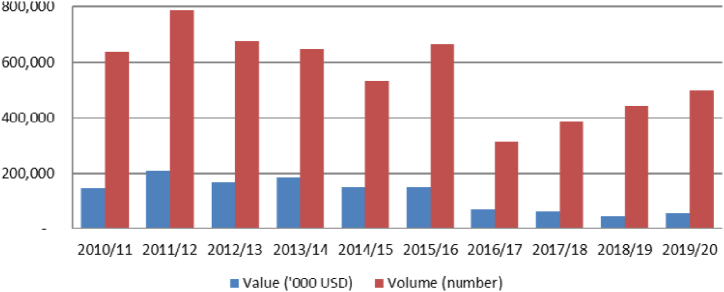
Trends in value and volume of live animal export.

Source: Rozina [10].

The trend of Ethiopian livestock exports from 2010/11 to 2019/20 is depicted in Fig. 2, which reveals a non-uniform trend over the years. Cattle, sheep, camels, and goats are the primary live animal species exported, with around half a million of each being sent abroad annually. These exports account for a total value of 123 million USD [10].

**Fig. 3:**
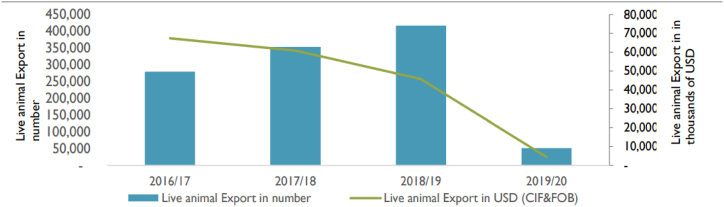
Ethiopian live animal export performance.

Source: FTFE-VCA [17].

According to FTFE-VCA [17] indicated a decline in both the number and value of the formal trade of live animal exports, where 87 % decline from 416,454 heads in 2018/2019 to 51,587 in 2019/20 in volume and 90 % decline in value in 2019/20 are recorded, as shown in Fig. 3 below. Yemen ranked first, with 60 % of the total export value, followed by Somalia and Djibouti at 30 and 5 %, respectively. Ethiopia Exports of live animals was US$27.13 Million during 2022 [25]. However, Ethiopia has the potential to increase the volume and value of domestic and export sales of meat and meat products. This could be achieved by increasing meat exports, expanding the commercialization of livestock production and marketing, diversifying into other products, and boosting domestic consumption.

**Fig. 4:**
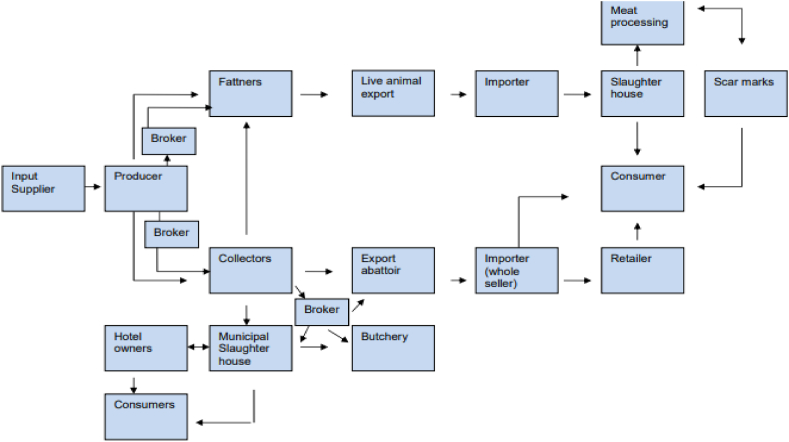
Value chain map for meat and live animals fatteners.

**Source:** Source: Jemal [8].

As indicated in Fig. 4, the general value chain for trading and exporting meat and live animals has historically not been a reliable, sustained relationship among actors within this value chain. Most relationships are casual and often change to suit the situation and the actors. Although value chain relationships work best when they are on a strict business basis, such relationships in the highlands can be characterized as clannish [17]. Although these relationships are not all clan-based, trust is built through such relationships, and being native to an area provides one significant advantage [23].

### 3.3 Meat animal value chain actors

Ethiopian meat and live animal value chains have developed over the years into a series of complex constituents involving various actors, including producers/farmers, collectors, small private and cooperative fatteners/feedlots, middlemen/brokers, livestock trading cooperatives, individual traders, and exporters [23].

According to Zekarias et al. [26], suppliers, producers, farmers, collectors, feedlot operators, traders, cooperatives, brokers, abattoirs, butchers, processors, and consumers are the major actors in the live animal and meat value chain in Ethiopia confirmed by various studies. Thus, the livestock market is structured so that the marketable livestock from the major producing areas reach the final consumer or end-user, passing through complex channels along the supply chains involving various actors [8,27].

In general, the major actors in the live animal and meat value chain in Ethiopia that were identified and confirmed by various studies are as follows:

**Input suppliers**: These are actors, among others, who are involved directly or indirectly in input supply for meat animal production, including supply of credit, cattle for fattening purposes, animal feed, veterinary services, training, and advisory services [28].

**Producers/Farmers**: Though the largest share of meat and live animals for export are produced by lowland pastoralists, they are involved in fattening different age categories of cattle for a limited period of time usually 3–6 months and finally supply for selling when the cattle are conditioned [11].

**Collectors**: These important market agents collect animals, usually from remote locations, and gather animals in the producer areas where watering points are found [29].

**Feedlots**: Feedlot/fattening operations include small-scale private feedlots and those that operate larger facilities aimed at animal export. They fatten cattle using traditional fattening systems that utilize available feed resources, mostly in semi-intensive feeding systems [8,20]. Private feedlot operators, import agents, animal health inspectors and development partners. Main activities are aggregation, quarantine, inspection, transporting and fattening meat animals for about 3 months [20].

**Brokers/Middlemen**: These are market agents that link buyers with sellers, mediate negotiations, and facilitate exchange terms [23]. In some market areas, particularly in remote rural locations, brokers not only provide important services but are also critical links to the markets for smallholders [8,20].

**Traders**: Both animal traders buy on average 100 animals per week, and small traders (usually buying on average 15 animals per week) in the market [29].

**Cooperatives**: Livestock cooperatives are located throughout the livestock production areas in Ethiopia; however, few exist in the highland areas [11,20].

**Live Animal Exporters**: Exporters collect animals from secondary markets (large and small traders, livestock trading cooperatives, collectors, and producers) [8,27,30].

**Abattoirs/butchers**: The export abattoirs all have networks in destination markets through which they sell their products. Butchers usually purchase fattened beef cattle from producers, farmers' traders, and small traders to slaughter at abattoirs and provide the same part of beef for hotels and restaurants and the other for local consumers at a specified place. They provide usually for customers in the form of Ethiopian roasted meat ‘tibs’ and raw beef ‘kurt’ [7].

**Hotel and restaurant owners:** These are the final actors in the value chain. Hotels are supplied with carcasses according to their specific requirements by butchers, while individual consumers buy carcasses directly from any butcher shop that satisfies their needs. They sometimes buy beef cattle from producers, farmers, and small traders through broker interference. They slaughter beef cattle at abattoirs, cook them, and serve them as meals to their customers [23,27].

**Consumers**: These are the final users who buy beef directly from butchers or value-added beef products from hotels and restaurants or who buy beef cattle either from producers or farmer traders to slaughter and share beef during holidays/religious-based ceremonies, festivities, and other informal institutional occasions [28,30,31].

### 3.4 Efficiency of meat animal transactions along the value chains

Ethiopian livestock marketing is characterized by poor market infrastructure and technical knowledge of value chain actors, inadequate market information, and poor linkages among actors [20]. Each stakeholder in the chain has a link to the next to form a viable chain by increasing productivity by improving quality, efficiency, and product differentiation [12,32].

In the Ethiopian live animal and meat marketing chain, individual actors perceive only for their benefit because the production mapping is not understood between actors [20]. Most producers fetch too old animals after being culled from production and those that might be abnormal due to disease and stress [19]. Traders transport animals using non-dedicated vehicles and endure long treks without providing feeding or watering for the animals [8,27]. The abattoirs in Ethiopia are poorly equipped [33]. In addition, butchers in Ethiopia often display meat on open shelves or unpack it, exposing it to dust, flies, mold, and unsanitary conditions [34].

As noted by Eshetie [16], unresponsive engaging of multi-attitude business linkages that severely affected the livestock value chain in Ethiopia. Hence, the sectors need professional and policy interventions to transport reliable and sustainable live animals and their products across market signals from the producer to the consumer. This is important in determining efficiency in the value chain through different efficiency assessments like Failure Mode and Effects Analysis (FMEA) method [32]. This method being a systematic, proactive tool for evaluating a process to identify where and how it might fail and to assess the relative impact of different failures, to identify the parts of the process that are most in need of change [35].

Government intervention in the value chain can significantly enhance vertical relationships among operational actors, as well as horizontal relationships with logistics providers. Additionally, such intervention can support market promotion measures aimed at attracting foreign direct investment and importers, while helping to transform traditional animal husbandry practices into commercial enterprises [20]. Resource-poor actors often struggle to justify and afford investments in innovations, which is why they are often seen as ambiguous and fail to generate measurable benefits [27].

According to Mummed and Webb [33], carcass grading and classification system also helps to develop clearer market signals from the consumer to the producer, assist producers to market their stock more effectively, improve efficiency in transactions, promote retail sales by the marking grading information on meat and facilitate the development of any export markets by improving communication between producers, traders, and consumers [19,32].

Generally, the efficiency of live animals and meat along the value chains in Ethiopian is poor and underdeveloped across the stakeholders as indicated above. This malfunctioning linkage in the chain affected producers and/or the resource-poor actors and even the country by prohibiting them from benefiting worth [8,23]. Thus, it seeks to develop a well-defined strategy for live animal and meat value chain management and intervention by the government to benefit from the existing potential.

### 3.5 Challenges of meat animal value chains

#### 3.5.1 Poor animal breeding practices

The absence of a market-oriented production system results in inconsistent and uneven supply of animals to markets [36]. Pastoralists consider their livestock as a means of saving or capital accumulation as a result they sell their livestock when need arises for cash income or when shortage of feed and water occurs [10].

Indigenous breeds despite their resilience and adaptation to local conditions, many indigenous Ethiopian cattle breeds, like the Borana and Ogaden, have smaller body sizes and slower growth rates compared to improved breeds [6,37]. This results in carcass weights 20–30 % lower, significantly impacting export competitiveness. Furthermore, suboptimal feed practices and inadequate disease control associated with poor breeding practices often lead to compromised meat quality. This includes lower fat content, tougher texture, and reduced shelf life, further hampering market access [6].

Although introducing improved breeds through crossbreeding holds promise, haphazard practices often lead to inconsistent results. Lack of access to quality breeding stock, poor record-keeping, and uncontrolled mating limit the effectiveness of crossbreeding programs, hindering genetic improvement [37]. Additionally, insufficient investment of public and private sector investments in breeding research and development are limited, impeding the development of tailored breeding programs suited to diverse Ethiopian contexts and market demands [11].

#### 3.5.2 Poor market infrastructure

Ethiopia's estimated 120 official livestock market centers are woefully inadequate. Most lack basic amenities like proper fencing, shelters, watering and feeding facilities, and quarantine zones [38]. The primary, secondary, and tertiary animal markets are poorly organized and fragmented, resulting in inadequate access to essential infrastructure such as holding grounds and water sources. This issue contributes to the low quality of livestock. Additionally, there is a significant lack of necessary slaughtering facilities, with few cold chain systems in place and a scarcity of modern abattoirs that comply with international hygiene standards [8,22]. Furthermore, high transportation costs in livestock trading often necessitate that animals are walked to markets and slaughterhouses, leading to considerable weight loss, physical injuries, and illnesses that can sometimes result in the death of the animals [19]. Consequently, these limitations pose significant challenges to animal welfare, product quality, and ultimately, market competitiveness. For example, Management Entity [39] highlights that investing in cold storage facilities and transportation infrastructure can greatly mitigate post-harvest losses while ensuring the quality of meat products. Enhancements to transportation systems and the establishment of efficient cold storage facilities will facilitate timely delivery of meat products, thereby reducing transaction time and costs. Although some initiatives, such as the Livestock Market Development project funded by the World Bank, have improved livestock markets and infrastructure [38] critical gaps persist [40].

There is a continued need for improved road networks connecting production areas to markets, increased use of information and communications technology (ICT) and media to relay market information and the construction of holding grounds with feed and water. Additionally, enhanced animal health services and grading standards at markets are necessary. Supporting private-sector abattoirs and meat-processing companies could also foster export competitiveness [19]. Addressing these significant infrastructure and institutional bottlenecks is essential to modernizing and transforming the livestock marketing system in Ethiopia.

#### 3.5.3 Illegal market value chain

The illegal marketing system for live animals and meat in Ethiopia is dominated by small farmer exporters and traders. Most livestock sales are related to farm households' cash needs and commercial orientation [41]. The supply originates in small numbers from highly dispersed small producers that supply non-market-oriented production systems [10]. The main challenges of the illegal marketing system are the absence of market information, absence of promotional efforts, sustainable supply problems, transport problems, prevalence of diseases, illegal export, inadequacy of infrastructure, competition, repeated bans, and inadequate port facilities [42].

Ethiopian livestock marketing is characterized by poor market infrastructure, inadequate market information, and poor linkages among value chain actors [9]. Small farmer exporters and traders are the major actors in the illegal cattle marketing system, while big traders and butchers transact larger numbers of mainly slaughter-type animals. Meat reaches consumers through a different channel and a different set of traders/businesses. This poor value chain development could plague the profitability of the actors [8]. Only a small proportion of animals are sold through official markets, with informal markets and direct trekking of animals from pastoralists to terminals accounting for many sales. For instance, in a study of cattle marketing in the Borana pastoral system, only 14 % were sold at primary markets [41]. This heavy reliance on informal networks undermines market transparency, competitive pricing, and value-addition possibilities.

#### 3.5.4 Illegal cross border trade

There is informal marketing of cattle, sheep, goats, and camels at border areas with Somalia, Kenya, Sudan, and Djibouti where the annual outflow of live animals from Ethiopia to neighboring countries via these routes is very high and the country loses a huge amount of foreign earning [10]. The share of informal cross-border trade from the total cross-border trade among neighboring African countries might be even disproportionately larger than the share of the formal economy [42]. Around 90 % of the cross-border trade along five Eastern African borders, including Ethiopia-Somaliland, Southern Somali-Northeastern Kenya, Western Ethiopia-Eastern Sudan, Southern Ethiopia–Northern Kenya and Northern Uganda–Southern Kenya, are the informal cross-border trade [43].

According to a study conducted by the Ethiopian Ministry of Trade, close to 1200 animals are illegally traded across the Ethiopian border every day. The study also estimates that the country loses 26.56 million US dollars annually due to the illicit trade. Also, Wassie [24], states that the informal cross-border livestock trade accounts for an estimated 71 % of the total value of live animal exports and 78 % of consumer goods and productive inputs imported into the Ethiopia-Kenya border area.

The trend of cross-border trade in the Horn of Africa, though generally increasing over time, has shown significant volume fluctuations and changes in patterns [42]. The high transaction costs of export in terms of time and money due to excessive regulations involving several ministries and agencies and related fees also contributed to the high level of informal trade while limiting the growth of formal trade across the international borders of Ethiopia [43].

#### 3.5.5 Lack of market information

Ethiopia, endowed with the largest livestock population in Africa, paradoxically struggles to optimize its livestock sector's potential. A critical factor impeding this trajectory is the pervasive lack of reliable, accessible market information within the live animal and meat value chain. Ethiopian market actors, encompassing farmers, traders, and processors, navigate a complex value chain obscured by inadequate market information [42]. Prices fluctuate erratically, furled by speculation and rumor rather than actual data on supply, demand, and animal quality. This opacity leaves farmers vulnerable to distress sales at significantly below-par prices, undermining their livelihoods and discouraging market participation [22].

Producers, traders, exporters, and support-giving institutions are constrained by a shortage of market information to rely on for enhancing production, marketing, and exports. The limited access to information (market prices, quality requirements, standards, etc.) reduces their ability to be competitive and to access profitable markets [19]. Hence, producers depend on actual market-day information obtained from relatives, friends, or neighbors for prices and selling decisions [44]. For consumers, the situation is equally opaque, fostering uncertainty about product availability and price variations, ultimately impacting their purchasing power and trust in the market system [18,20].

The consequences of this information vacuum extend far beyond immediate price fluctuations. The lack of transparency breeds inefficiency and inequity, creating breeding grounds for exploitative practices by middlemen and brokers [45]. These actors capitalize on knowledge asymmetries, acting as information gatekeepers and extracting exorbitant profits through opaque transactions. Furthermore, the absence of readily available data on animal health, carcass weights, and grading standards impedes effective quality control and traceability, risking food safety and hindering market competitiveness [39].

One opportunity to enhance transaction efficiency is through the digitalization of transactions within the meat animal value chain. the adoption of digital platforms can facilitate real-time information sharing and reduce transaction costs. By using digital platforms stakeholders such as farmers traders and processors can connect directly eliminating intermediaries and reducing transaction costs [20].

#### 3.5.6 Lack of slaughter-ready animals

The inadequate supply of quality cattle and small ruminants meeting slaughter criteria severely constrains Ethiopia's meat value chain [24]. Several interlinked factors drive this shortage. Subsistence-oriented production dependent on seasonal conditions engenders inconsistent market off-take and supply fluctuations [37]. The predominant use of local breeds with limited adoption of genetically improved stock or strategic crossbreeding curbs productivity. Additionally, poor husbandry and limited veterinary services precipitate diseases and malnutrition, depressing animal growth rates and fitness [9]. Moreover, weak coordination amongst actors inhibits demand-driven production planning tailored to abattoir requirements [24]. This leaves producers without incentives to supply targeted slaughter animals. The lack of well-defined grade standards for classifying animal age, weight, and conformation allows the sale of immature and unfinished stock [14]. Transaction opacity arising from information gaps further hampers supply responsiveness [45].

To enhance the availability of slaughter-ready cattle and small ruminants, integrated strategies are imperative across multiple domains. Production planning incentives can align outputs to slaughter criteria and specifications [46]. Upgrading animal health services, genetics, and feeding systems is also critical. Infrastructure investments into holding grounds, watering points, and abattoir access can enable animal aggregation and delivery. With appropriate coordinated interventions spanning inputs, practices, transactions, logistics, and facilities, a steady supply of quality animals meeting industry needs can be achieved across the meat value chain.

#### 3.5.7 Lack of grading and quality audit systems

Grading and quality auditing of meat is important to ensure food safety, consistent quality, and fair pricing. However, there is a lack of robust grading and auditing systems in many countries and regions [29]. This includes developing countries where smallholder livestock production dominates, as well as some developed countries with fragmented supply chains like the United States [14].

Similarly, absence of a comprehensive grading and quality audit system constitutes a major challenge across Ethiopia's meat value chain. Standards and procedures for systematically classifying and certifying the quality of live animals and carcasses are not implemented widely. This hampers transparency, efficiency, and product differentiation [29]. For instance, the dark cutting, improper handling of the product, poor sanitation, careless packing and poor management during transport [47] are also the problems of meat hindering Ethiopia's competitiveness in the region.

According to some studies, key factors inhibiting the development of grading and auditing systems include.

• Lack of incentives and capacity building for producers and processors to adopt rigorous classification practices. Compliance costs tend to disadvantage small-scale actors [29,47].
• Infrastructural limitations, as grading requires proper holding facilities, chilling technology, and sanitary slaughter processes which are inadequate currently [39].
• Insufficient institutional coordination between various regulatory agencies to formulate and enforce a harmonized grading framework [24,48]. For instance, grading is conducted differently across countries like the U.S. relies heavily on voluntary quality grading, while the EU and UK have mandatory carcass classification schemes. This contributes to inconsistent grading worldwide [49].
• Absence of consumer awareness regarding quality differentiation, muting demand-side drive for rigorous grading [20].
• High subjectivity and cost of manual grading methods, coupled with lack of technical skills in new instrumental techniques [11].
• Prevalence of informal transactions where grade classifications hold little value [24,29].

Recent studies show substantial opportunities to improve grading and auditing. For instance, enhanced carcass grading to reward quality could increase the value of U.S. beef output by $1.2 billion annually. Meanwhile, adoption of new camera and computer vision technologies can help enable more accurate, automated grading [49].

The absence of a comprehensive grading and quality audit system constitutes a major challenge across Ethiopia's meat value chain, as noted previously. The inability to demonstrate verified quality grades inhibits access to higher value overseas markets for meat products. As such, live animal shipments dominate exports despite lower per unit value [50]. In addition, the absence of auditing further hampers the transfer of improved technologies and processes from the export arena into the domestic meat value chain [47]. Strengthening grading and certification systems can thus catalyze a shift from live animal exports towards higher-value processed meat products [14,29].

Therefore, appropriate grading frameworks attuned to diverse Ethiopian contexts will enable actors across the system to signal and verify quality attributes expected by overseas buyers. Such signalling can expand export opportunities and revenues, while disseminating best practices into domestic markets [24].

Additionally, to enhance grading and auditing, efforts are needed to build capabilities across the system, upgrade infrastructure, implement decentralized and transparent processes, catalyze consumer demand for quality verification, and improve coordination between stakeholders. Furthermore, developing clear national grading standards, training graders, implementing audits, and using technologies to reduce human error and subjectivity. Government and industry collaboration is needed for systemic improvement [23]. Specially, grading systems tailored for the Ethiopian context can drive competitiveness, efficiency and food safety within the meat value chain.

### 3.6 Prospects to strengthen the meat animal value chain

The prospects exist to enhance efficiency, competitiveness and inclusivity across Ethiopia's meat value chain. Ongoing efforts seek to upgrade infrastructure, improve market information systems, strengthen relationships between value chain actors, promote quality standards, and developing new meat processing facilities [20]. For instance, the Livestock Master Plan aims to modernize production through improved breeds and technology, expand extension services, and enhance domestic and export abattoirs to bolster the legal marketing system [50]. Concurrently, integrating digital tools to share pricing data and formalizing cross-border trade through regulations and incentives can curb harmful illegal practices [43]. If supported by appropriate policies and public-private investment, such measures can help optimize benefits across the meat value chain for Ethiopian producers, traders, processors, and consumers.

#### 3.6.1 Infrastructure upgrades

Ongoing infrastructure initiatives aim to strengthen physical livestock marketing facilities and logistics networks. For instance, the World Bank-funded Ethiopia Livestock and Fisheries Sector Development Project has established and upgraded cattle market centers, holding grounds, and loading ramps in major production areas like Borena [40]. Further investments in road, rail, cold chain, and IT infrastructure could strengthen animal transport and connectivity between nodes in the value chain. However, questions remain regarding the financial sustainability and inclusivity of such top-down interventions [45]. Impact assessments are needed to ensure infrastructure upgrades beef access to market information digital platforms and media broadcasting show promise for expanding producers' access to pricing data and buyers [51]. Yet broader outcomes analysis is required, as information asymmetries likely still disadvantage smallholders during transactions. Sustainable business models for providing accessible, timely market information remain elusive.

#### 3.6.2 Coordinating value chain actors contract

Farming and out grower schemes attempt to align producers, processors and exporters through vertical coordination [8]. But power imbalances continue to privilege large downstream players, to the detriment of farmers [48]. Stronger institutional support is essential to ensure equitable gain distribution along the chain.

#### 3.6.3 Promoting standards ongoing

The promotion of animal traceability, welfare, sanitary, and meat grading standards has the potential to enhance quality and food safety [33]. However, adoption levels remain low, signaling a need for tailored capacity building and incentives to facilitate wider compliance across heterogeneous smallholders [29].

#### 3.6.4 Developing processing facilities

Meat processing and export abattoirs hoped to absorb domestic surpluses, raise value addition, and enhance smallholder inclusion [50]. Unfortunately, links to small-scale suppliers are weak, limiting trickle-down benefits. Impact assessments around processing facilities are vital [29]. a nuanced, system-wide approach is essential to ensure meat value chain improvements translate to inclusive, pro-poor gains rather than exacerbating structural inequities.

In addition, Ethiopia's strategic location is advantageous for transporting fresh products within a short period, especially to Middle East markets. This, coupled with the country's vast livestock resources, labor force, and abundant water resources, can be leveraged to boost the meat animal value chain [52]. the country is also experiencing an increase in domestic and foreign demand for meat and live animal products. This demand is driven by population growth and changing dietary preferences, which presents a significant opportunity for the growth of the meat-animal value chain [8,29]. However, it's important to note that while these opportunities exist, they can only be fully realized if the challenges facing the meat and live animal value chain in Ethiopia are addressed and must be backed by enabling policies and coordination.

### 3.7 Efforts to improve the animal meat marketing system

The Ethiopian government and development partners have undertaken various initiatives to address challenges and transform the livestock marketing system. Efforts to improve the marketing system in Ethiopia include initiatives to enhance market information, address market constraints, strengthen relationships among value chain actors, promote quality and standards, develop new meat processing investments, and explore market opportunities and Ethiopian competitiveness [20]. However, critical gaps remain in fully realizing intended outcomes. Some of the key efforts and opportunities for improvement are:

Market information systems have been promoted through platforms like the Livestock Market Information System (LMIS) and the use of ICT and media broadcasting [18]. However, Studies find persistent knowledge and power asymmetries disadvantaging smallholders, despite greater pricing transparency [20,45]. Sustainable business models for equitable market information provision are yet to emerge.

Contract farming and out-grower schemes attempt vertical coordination along the chain [15]. often, agreements frequently favor large firms, with limited gain sharing to small-scale producers [48]. More institutional support is needed to ensure equitable contractual arrangements.

Standards promotion around traceability, welfare, and grading have been pursued, but adoption remains low. Significant heterogeneity amongst smallholders inhibits compliance, signalling a need for tailored capacity building [5]. Also, upgrading livestock markets and logistics infrastructure has been a policy priority. However, the lack of pro-poor targeting risks excluding marginalized groups from the intended benefits of interventions [13,45]. Improving inclusivity in infrastructure projects is critical.

The government has also introduced a new law to regulate the livestock sector, which will increase its efficiency and value by eliminating the black market. The new law will require all traders to be licensed, all animals to be registered, and all livestock markets to be identified either as primary or secondary [53]. Regional and city authorities will administer auction markets, while any transaction of livestock outside of unlicensed markets will be illegal under this law [53].

Recent initiatives have analyzed export opportunities suited to strengths in Ethiopia's meat value chain, along with barriers inhibiting competitiveness. Shapiro et al. [50] indicated that there is still considerable unmet demand in Middle Eastern, East African, and East Asian markets, especially for chevon and mutton products. However, inconsistencies in quality, volume and logistics hamper export development. To address this, investments into breeding programs tailored for target export markets are recommended [10]. In addition, aligning slaughter-ready animals with importers' specifications can improve price competitiveness. Furthermore, certification systems verifying origin, traceability, standards, and food safety are also critical for high-value trade channels [15].

Infrastructure upgrades in transport and logistics could enable large-scale reliable supply to export hubs. Nonetheless, equitable participation is crucial - export-driven changes must not marginalize smallholders through structural barriers. With appropriate safeguards, analysis-guided strategies hold potential to grow high-value trade. But inclusive mechanisms are vital for broad, sustainable gains across the chain [48].

## 4 Conclusion

Ethiopia's livestock sector holds immense potential, boasting the largest cattle population in Africa. However, the efficiency of meat animal transactions across the value chain remains severely underdeveloped. A multitude of challenges hindered the system, ranging from poor infrastructure and lack of market information to absence of grading practices and illegal cross-border trade. These factors culminate in inefficient transactions that disproportionately disadvantage producers and resource-poor actors.

Complex value chain involving a diverse array of stakeholders-input suppliers, farmers, traders, cooperatives, exporters, processors, retailers and consumers. However, weak linkages and misaligned incentives among this chain undermine connectivity and value addition. Live animal exports overwhelmingly surpass meat product exports due to supply constraints, inability to meet quality standards, and lack of aggregation systems tailored to export requirements.

Despite the prevailing challenges, opportunities exist to strengthen Ethiopia's meat value chain. Ongoing efforts aim to upgrade infrastructure, digitize market information systems, promote quality assurance through grading and certification, align production planning with export demands, and attract investments into meat processing. Legislative reforms to formalize livestock marketing channels also hold promise. However, technical interventions alone are insufficient. A coordinated, systemic approach backed by inclusive policies and equitable power dynamics is crucial. Empowering smallholder producers, building sustainable market linkages, and distributing gains across the supply chain must be prioritized. Only through such holistic strategies can Ethiopia harness the full socioeconomic benefits of its vast livestock resources.

Enhancing efficiency in meat animal transactions requires commitment from all stakeholders - government agencies, private enterprises, development partners and crucially, the producers themselves. With concerted multi-stakeholder efforts, Ethiopia can progressively modernize its livestock marketing system, bolster export competitiveness, ensure food security, and uplift livelihoods across rural communities. Unlocking these transformative impacts is vital for sustainable economic development.

## CRediT authorship contribution statement

**Asrat Ayza Wakaso:** Writing – review & editing, Writing – original draft, Methodology, Conceptualization. **Yesihak Yusuf Mummed:** Writing – review & editing, Conceptualization. **Yonatan Kassu Yesuf:** Writing – review & editing, Formal analysis.

## Data availability statement

The raw data supporting the conclusions of this article will be made available by the authors, without undue reservation.

## Declaration of competing interest

The authors declare that they have no known competing financial interests or personal relationships that could have appeared to influence the work reported in this paper.
